# Treatment of a Case of Cerebro-Spinal Meningitis

**Published:** 1885-05-20

**Authors:** J. McF. Gaston

**Affiliations:** Professor of Principles and Practice of Surgery in the Southern Medical College, Atlanta, Georgia


### Southern Medical Record.

EDITORS:

THOMAS S. POWELL, M. D.    R. C. WORD, M. D.

*R. C. WORD, M.D., Managing Editor.*

☞All Communications and Letters on Business connected with the RECORD must be addressed to the Managing Editor.

VOL. XV.    ATLANTA, GA., MAY 20, 1885.    No. 5.

### ORIGINAL AND SELECTED ARTICLES.

### TREATMENT OF A CASE OF CEREBRO-SPINAL MENINGITIS.

By J. McF. GASTON, M. D.,
Professor of Principles and Practice of Surgery in the SOUTHERN MEDICAL COLLEGE, Atlanta, Georgia.

On the 18th of April, 1885, I was called out some five miles on the McDonough road to the house of Mr. —— Bowers, to attend the little son of Mr. T. C. McLendon, called Lester, and three and a half years old. There was great heat of the head, with flushed face and dilatation of the pupils, while the general surface of the body and the lower extremities were not above the natural warmth, and there was a mottled appearance of the skin. The mother stated that there had been, previously, red splotches over the surface, which had disappeared under the use of warmth and moisture, with a sheet wrapped about his body, under the direction of a water-cure adviser who had been called in to see the case. The pulse was 100 beats to the minute and full, with marked tension and regularity. There was writhing of the body, with repeated sharp outcries, without consciousness of what was said to him, and without any articulate expression at any time during the hour spent with him. He had been sick for two days previously.

The unrest and tossing of his arms and legs, with a fixed stare of the eyes, accompanied by the entire want of mental perception, indicated one of the forms of cerebro-spinal meningitis; and, accordingly, I entered upon a mode of treatment which seemed to promise better results than has usually attended the therapeutic measures in this very grave affection. Viewing this disease, in its outset, as connected with an impairment of the nerve-centers, dependent upon derangement of the biliary function, and partaking of the nature of pernicious congestive disorders, the correctives used were as follows:

The little patient was placed in a tub of warm water, with a blanket wrapped around his shoulders and extending over the tub so as to keep in the warm vapor, hot water being added when the temperature diminished. A handful of powdered mustard seed was added to the water at the outset. Cold water was applied, with cloths, to his head during the entire half hour that he remained in the warm bath. In the meantime he was given a powder of the following prescription:

℞ Sulphate of quinine, } aa........................grs xii.

Saccharated calomel }

Divided in 4 powders; one to be taken every two hours.

Diet of chicken water.

It was only by prizing his mouth open with a large spoon, and passing the medicine into his throat in a teaspoon, that it could be given then or subsequently during the day. A mustard plaster was applied along the spine.

There was a partial restoration of the equilibrium of the circulation from the application of warmth to the body and cold to the head; which latter was directed to be kept up constantly by cloths wrung out of the cold water.

After taking the powders, direction was given for him to take the following:

℞ Salicylate of soda..................................ʒ j

Fluid extract of jaborandi, } aa....................℥ j.

Syrup of orange peel, }

Take a small teaspoonful every two hours.

The verbal instruction to mix it with a tablespoonful of water not being observed, the medicine was rejected by the stomach each time that it was thus forced down by the leverage of a large spoon as above-mentioned.

April 19th.—The combination of calomel and quinine had produced two evacuations from the bowels, heat of head lessened, and some return of consciousness, with a pulse of ninety to the minute, full and tense, with pupils still dilated.

There were some petechial spots near the knees, and well-defined retraction of the head.

The tongue was considerably coated, and there was evident thirst, as he now spoke in an incoherent manner, asking for water, but rarely swallowed any when put to his mouth.

The prescription of quinine and calomel was repeated and given as on the previous day, with directions to resume the other medicine after finishing the powders, diluting it with water.

April 20th.—The symptoms had undergone a complete change and there was no longer any undue determination to the head, while the pulse counted 110 to the minute, without that tension previously observed. The warmth of the surface was somewhat more than natural, corresponding, however, to that of the head and face. The bowels had been moved again after taking the calomel and quinine. The stomach had not rejected the salicylate of soda and jaborandi mixture when diluted, and this was continued every 2 hours.

April 21st.—The moderate reaction of yesterday had given way to a state of prostration, and the little patient lay in a semi-comatose condition with his mouth partially open, and upon raising the eyelids, which were closed, the pupils were found contracted to less than the normal size. He was ordered a whisky toddy, and, upon being raised by his father to drink it, a striking and extraordinary dilatation of the pupils occurred, manifesting the lack of a controlling nerve element in this contrast within such a brief space of time. The warmth of the surface was now below the natural state, extending throughout every part, and even to his face and ears.

The pulse was 130 to the minute, and without resistance upon pressure. There were scarcely any traces left of the ecchymosed spots, and the opisthotonos had diminished very much.

He passed urine in the bed, without asking to be taken up, as he had done on the previous day upon desiring to make water.

He was ordered a tablespoonful every two hours of a solution of carbonate of ammonia, containing 20 grains to the ounce of water, and to take a tablespoonful of whisky in the interval made into punch with fresh sweet milk, with mustard plasters to his extremities.

April 22d.—The tendency to collapse seemed to be arrested, and his mental condition had improved, so that he drank the milk punch and took his medicine without making resistance.

The pulse was 120 to the minute, with more tone, and the natural warmth had returned to the surface, while the tongue was clean and moist. He was given 1½ grains of quinine every four hours, continuing the carbonate of ammonia and milk punch. The bowels had moved and urine was passed in the bed, without asking to be taken up. Poultice with Peruvian bark applied to the abdomen.

April 23d.—The patient had evidently improved in all respects, and though the pulse was 125 to the minute, it was not so feeble as for the past two days. He was directed to take a teaspoonful of Colden's liquid beef tonic every four hours, and continue with the carbonate ammonia and milk punch in the intervals. The poultice with Peruvian bark was replaced by a flannel with spirits turpentine and camphor to the bowels.

On the 24th he was not seen, and on the 25th it was found that his bowels had been too frequently moved, with mucous discharges and considerable tympanitis. The pulse was 120 to the minute, and the tongue free from any coating, but somewhat redder and dryer than natural. He was ordered the following mixture:

| | | |
|---|---|---|
| ℞ | Spts. turpentine | fl. ʒ j |
| | Carbonate of ammonia | ʒ j |
| | Elixir of paregoric | fl ʒ ij |
| | Camphor water, } aa | fl. ℥ iij. |
| | Mucilage of acacia, } | |

Mix and take half a tablespoonful every two hours.

Stop the beef extract, and make the whiskey punch with boiled milk, to be given after each dose of the medicine. Continue the application of spirits of turpentine and camphor to the bowels.

April 26th.—Discharges of mucous from the bowels frequent, and some tympanitis, with pulse of 140 to the minute, with tongue smooth and red, but not dry. His consciousness has returned, and he takes notice of what passes or what is said to him, protruding his tongue, etc.

In addition to the medication of yesterday the following is taken without coercion:

| | | |
|---|---|---|
| ℞ | Sulphate of quinine | grs. xij |
| | Acetate of lead | grs. vj |
| | Tannin | grs. iv |
| | Opium | grs. ij. |

Mix and divide into 12 powders.

Take one powder at intervals of 2, 3 or 4 hours, according to the frequency of the discharges from the bowels.

Apply a red-oak poultice to the bowels constantly, after rubbing the surface with the camphorated spirits of turpentine.

Whatever may be the final result of this case, it is evident that the symptoms indicative of the cerebro-spinal trouble were controlled by the use of the combination of calomel and quinine, followed by the salicylate of soda and fluid extract of jaborandi, and the subsequent intestinal irritation and tendency to a typhoid form of fever may be regarded as an accidental development and not as a sequel or consequence of either the disease or the treatment instituted. None of the ordinary results of aggravated cases, such as deafness or impairment of vision, connected with cerebral effusions, have occurred after the lapse of ten days since the access of the disease, whereas it has not generally been observed that intestinal troubles are a sequence of it.

April 27th.—Found that the irritation of the bowels had ceased after the second dose of the astringent powders, when, according to directions, they were suspended; and there had been no occasion to resume this class of remedies up to the hour of my visit late in the afternoon. The red-oak bark poultice was also discontinued, with directions to keep a flannel roller with camphorated turpentine around the abdomen. There is no longer any tympanitis; the tongue is not so slick and red as on yesterday; pulse 125 to the minute, and the previous night and this morning have been passed in a quiet state, most probably the result of the opiates.

The terebinthinate mixture was continued, alternating with one grain doses of quinine every 2 hours, and the milk punch to be taken after each regularly.

Although he still has some stiffness of the neck, the head is not drawn backwards as formerly, and while he sleeps frequently, it is not of a comatose nature, so that his condition is more promising to-day.

April 28th.—The night was passed in a restless state, and during the day he has manifested some agitation, but not accompanied with febrile disturbance. The pulse is 125 beats. The bowels have been moved twice only within 36 hours, and indicating less irritation in the character of the discharges. There is no tympanitis, and the tongue is moist, with some slight coating over the surface. Gave 5 grains of hydrate of chloral, with directions to repeat the same hourly for 3 times if requisite, and should quiet not be secured within 2 hours afterwards, to give $\frac{1}{4}$ grain of morphine. Continue the terebinthinate mixture and quinine, with the milk punches.

Notwithstanding that the stiffness of the neck continues to some extent, there is no indication of the impediment of hearing, nor of the inflammatory affection of either eye, as has often occurred in cases of meningitis. The intellectual faculties have not suffered any detriment from the disease.

April 29th.—Three doses of the solution of chloral were given last night without securing composure, and only after $\frac{1}{4}$ grain of sulphate of morphine did the patient sleep. After resting for three hours he roused up and was again giving signs of restlessness when I visited him at 2 o'clock p. m., so that the chloral was resumed with instruction to give it every 2 hours, and if he did not get quiet before midnight to repeat the morphine. The pulse was 130 to the minute, temperature 101°; the bowels had been moved only once, and that slightly; the pupils appeared normal, the tongue natural, and he manifested no mental disturbance, but had little disposition to take his milk punch. The terebinthinate preparation alternated with 1 grain of quinine, so that each should be taken every four hours, had been given regularly and was ordered to be continued, with the application of camphorated spirits of turpentine to his spine and back of his neck. The stiffness of the neck, with slight retraction of the head, has not entirely disappeared, but there are no other evidences of the local effects of the cerebro-spinal meningitis perceptible at present.

April 30.—The chloral was given repeatedly, with only partial composure, but the morphine was not given, and at the hour of my visit at 1 o'clock p. m. he was kicking and throwing his arms about with anxious outcries from time to time.

Dr. K. C. Divine saw the patient in consultation with me to-day, and, with a view to test his perception, repeated an experiment I had made in lighting a match to look at his tongue, when the little fellow put his lips together, and blew at it several times, while it was held at a distance, until he succeeded in extinguishing the light. This was repeated until we were satisfied that it was intentional. The pulse to-day is 130 beats to the minute; temperature, 100°; tongue clean; bowels have been twice moved, and he took one of the astringent powders. We stopped the chloral and directed morphine at night. Take 1 gr. quinine 3 times a day and continue the turpentine internally and externally. Milk punch and soft eggs to be given frequently, day and night.

May 1st.—I was called early this morning to visit the patient, with the statement that he passed a restless night, notwithstanding that the $\frac{1}{4}$ grain of morphine was given every four hours until three doses were taken. This quantity in a child of three and a half years was expected to be sufficient to induce a profound sleep, yet its effect was only shown by itching of the nose, and the inference is that the action of morphine is not suited to the case, so that other agents for calming the excitement must be used in the future.

Observing, from a small, ash-colored, fæcal evacuation, that there was a want of biliary discharge, I gave at once, upon arrival, 3 grains of saccharated calomel, with instructions to repeat the dose every four hours until the color of bile appeared in the passages.

Within less than two hours there was another evacuation of good consistence, which indicated a change in the color, but it is scarcely to be inferred that the calomel could have had this effect in so short a time.

Bromide of potash, 5 grains, was ordered every two hours, and in case composure was not obtained during the afternoon to add a like quantity of hydrate chloral, which being given in combination for several times and failing to secure rest, Hoffman's anodyne in doses of half a teaspoonful every hour was directed to be given after midnight, suspending all the other medicines. The milk punch was drank from a cup without any difficulty during my stay at the house, and he is to have it frequently.

The pulse was 125, temperature 100°, tongue clean and no longer any tympanitis, while the stiffness of the neck was slight. The pupils responded to the light and appeared ordinarily to be normal. Any attempt to control his tossing about was unavailing, so that there is evidently an impairment of the nerve-element which requires correction at the same time that the hepatic secretions demand attention.

